# Tricuspid Valve Vegetation Secondary to Ovarian Cancer Leading to Acute Stroke via Pulmonary Arteriovenous Malformation

**DOI:** 10.7759/cureus.17136

**Published:** 2021-08-12

**Authors:** Arafat A Farooqui, Rabiah Ashraf, Racquel D’Ornellas, Awais Aslam, Michael Marcelin, Vijay Shetty

**Affiliations:** 1 Internal Medicine, Maimonides Medical Center, Brooklyn, USA; 2 Cardiology, Maimonides Medical Center, Brooklyn, USA

**Keywords:** embolism, endocarditis, stroke symptoms, cancer, arteriovenous malformation

## Abstract

We report a case of non-bacterial thrombotic endocarditis in a 52-year-old woman due to ovarian cancer that was complicated by acute ischemic stroke through pulmonary arteriovenous malformation. Echocardiography showed tricuspid valve vegetation and a positive bubble study that revealed pulmonary arteriovenous malformation in the absence of patent foramen ovale. The patient opted for palliative management and was discharged home with comfort care.

## Introduction

Vegetations on cardiac valves are a common occurrence. They are mostly formed on previously damaged valves. When microorganisms are not identified as the causative agent, remaining etiologies such as existing malignancy or hypercoagulable states must be explored. These sterile vegetations are referred to as nonbacterial thrombotic endocarditis (NBTE). Vegetations have an increased propensity to embolize resulting in organ-specific symptoms. While there are studies documenting strokes resulting from left heart vegetations, strokes originating from right heart vegetations in the absence of a patent foramen ovale (PFO) are extremely rare.

## Case presentation

A 52-year-old Chinese woman with recently diagnosed stage IV ovarian adenocarcinoma with lung metastasis and chronic deep venous thrombosis (DVT) presented to the emergency room (ER) with fever, shortness of breath, generalized weakness, and confusion. Upon admission, she had a temperature of 100.9˚F, blood pressure of 113/54 mmHg, heart rate of 89 beats/minute and respiratory rate of 22 breaths/minute with oxygen saturation >94% on room air. Examination revealed left facial droop, left homonymous hemianopsia, left upper extremity and lower extremity weakness (2/5 and 2/5, respectively), left-sided sensory loss with an NIH stroke scale of 14. Initial differentials included acute pulmonary embolism (PE), acute ischemic stroke, and sepsis.

**Figure 1:**
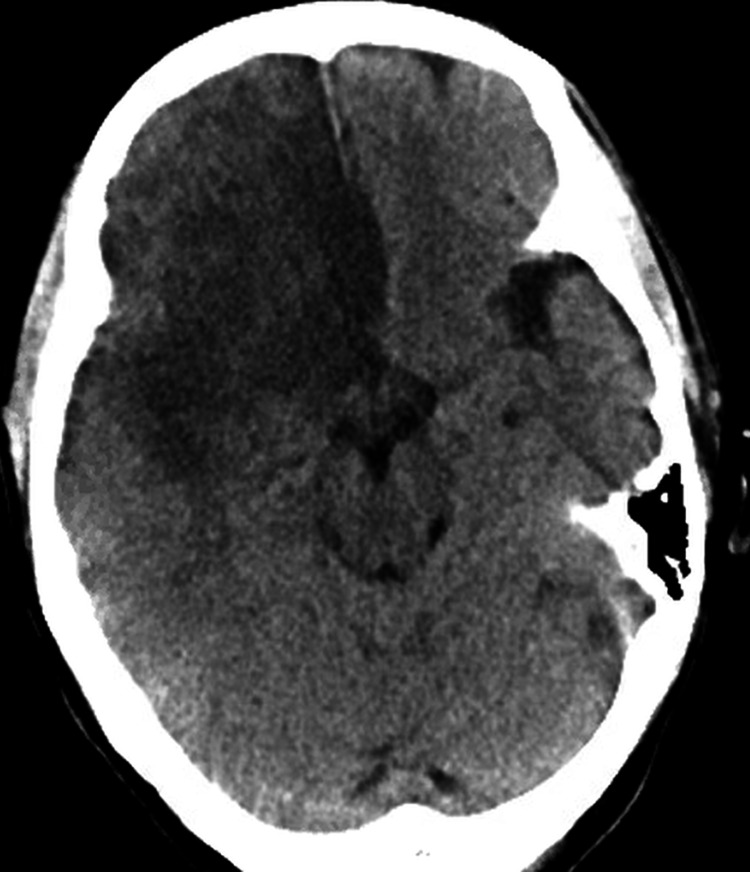
CT head showing right ACA and MCA stroke ACA: Anterior Cerebral Artery, MCA: Middle Cerebral Artery

Workup revealed leukocytosis of 41.2 K/μL (4.8-10.8 K/μL), with 87% neutrophil count (37.9%-70.5%) and bandemia of 19% (0%-8%). Hemoglobin was 8.1 g/dL (12-16 g/dL) with mean cell volume (MCV) of 93.8 FL (81-99 FL) and hematocrit of 26.2% (37%-47%). Urinalysis was negative for infection. The respiratory viral panel including COVID-19 was negative. Chest x-ray was also negative for any ongoing pathology. CT head (Figure 1) demonstrated large right anterior cerebral artery (ACA) and middle cerebral artery (MCA) stroke with hemorrhagic conversion and a 3 mm midline shift. CT chest confirmed acute PE in the lingular segmental branch and right lower lobe pulmonary artery. Upper and lower extremity venous duplex studies revealed no acute DVT.

Pan cultures (blood, respiratory, urine) consistently resulted in negative microbial growth, but antibiotics were continued due to persistent leukocytosis. There was a concern that clots in pulmonary vessels might have embolized to the brain through PFO resulting in a stroke. So echocardiography with bubble study was performed that showed focal thickening at the tip of the tricuspid leaflet, consistent with vegetation. A saline contrast study showed delayed appearance of bubbles in the left atrium that were arising from the left superior pulmonary vein. There was no PFO noted and findings were consistent with left upper lobe pulmonary arteriovenous malformation (AVM) (Videos 1, 2).

**Video 1:** Apical four-chamber view showing tricuspid valve vegetation

 

**Video 2:** Bubble study showing a delayed appearance of bubbles in left atrium through pulmonary AVM AVM - arteriovenous malformation

In the combined setting of echocardiography findings with multiple negative blood cultures, persistent fever while on antibiotics, and increasing leukocytosis; the diagnosis was finally revised to NBTE secondary to known ovarian malignancy. Acute embolic stroke was attributed to embolization of tricuspid vegetation through pulmonary AVM. Anticoagulation was contraindicated due to hemorrhagic conversion of the stroke. The patient opted for palliative management and was discharged home with comfort care.

## Discussion

NBTE is characterized by the deposition of fibrin and platelet micro-thrombi on relatively normal heart valves in the absence of bacterial infection in the blood. It is a potentially underdiagnosed sequel of the pro-thrombotic state noted in malignancy. Different pro-thrombotic states can result in NBTE, with malignancy and autoimmune diseases being the most common causes [1]. Since all cardiac vegetations have a high propensity to embolize, NBTE most notably can lead to recurrent or multiple ischemic cerebrovascular strokes [2]. In the case described in this manuscript, the most likely cause of NBTE is ovarian malignancy. It is still unclear how NBTE actually arises but different mechanisms have been postulated including (1) release of tumor cell-derived cytokines, (2) direct tumor cell-endothelial cell interactions, and (3) release of tissue factor and cancer pro-coagulant by cancer cells, expression of urokinase-type and tissue-type plasminogen activator and release of ADP, thrombin, and other proteases [2].

Biller et al. [3] reported aortic valves as the most commonly affected valves in NBTE followed by mitral and then right-sided heart valves. Embolization of right-sided cardiac vegetations is most commonly seen in intravenous (IV) drug users via PFO. In most of the reported cases of endocarditis, there is some degree of systemic embolization that was associated with the presence of a PFO [4,5]. In this case, the contrary was noted, with the tricuspid valve being the singular valve involved with embolization to the brain - in the absence of both IV drug use and a PFO.

High clinical suspicion and timely use of echocardiography carry paramount importance in diagnosing NBTE to establish the existence of vegetations. In our patient with consistently negative blood cultures and associated negative septic workup, the leukocytosis, bandemia, and persistent fevers were attributed to malignancy. Leukocytosis was also attributed to the use of steroids for normalization of intracranial pressure in the setting of hemorrhagic conversion of stroke. Of note, echocardiography, in this case, revealed no PFO; however, some studies have shown to be the conduit for these vegetations to embolize to the brain. The delayed appearance of bubbles in the left atrium (>6 cardiac beats) on saline contrast echocardiography is diagnostic of pulmonary AVM [6] as shown in video 2. The early appearance of bubbles is diagnostic of PFO. To the best of our knowledge, this is the first case reporting NBTE complicated by acute stroke via pulmonary AVM.

The incidence of NBTE is unknown. Patients with adenocarcinoma between the fourth and eighth decades of life with uncertain sex distribution were found to be at higher risk. Clinical manifestations of NBTE vary and there are no pathognomonic features specifically attributed to NBTE. Cardiac murmurs are infrequently noted in NBTE [7]. If present, these soft systolic murmurs are located at the left lower sternal border and are nonspecific. However, if a new murmur is present in a known cancer patient, a diagnosis of NBTE should be considered. Almost half of the patients with NBTE present with systemic embolization. Lopez et al. [8] reported the varied incidence of systemic emboli with NBTE as 14%-91%. The most common circulations affected are the cerebral, coronary, renal, and mesenteric circulations. Out of these circulations, the most common clinical presentation of NBTE is a sudden neurological deficit [2].

The primary treatment of NBTE is source control; the control of malignancy and the use of anticoagulants to decrease the likelihood of recurrent thromboembolic episodes [2]. Treatment options for pulmonary AVM include percutaneous catheter-based closure, endovascular embolization, or surgical closure.

## Conclusions

NBTE should be considered a possible diagnosis in cancer patients when patients present with sudden neurological deficits. This manuscript highlights a rare diagnosis of embolic-ischemic stroke in a patient with advanced ovarian carcinoma resulting from NBTE through pulmonary AVM in the absence of PFO.

## References

[1] Ferrans VJ, Rodríguez ER (1985). Cardiovascular lesions in collagen-vascular diseases. Heart Vessels Suppl.

[2] el-Shami K, Griffiths E, Streiff M (2007). Nonbacterial thrombotic endocarditis in cancer patients: pathogenesis, diagnosis, and treatment. Oncologist.

[3] Biller J, Challa VR, Toole JF, Howard VJ (1982). Nonbacterial thrombotic endocarditis. A neurologic perspective of clinicopathologic correlations of 99 patients. Arch Neurol.

[4] Johri AM, Kovacs KA, Kafka H (2009). An unusual case of infective endocarditis: extension of a tricuspid valve vegetation into the left atrium through a patent foramen ovale. Can J Cardiol.

[5] Yousef GM, Okhumale PI, Aljoudi H, Cansino S (2017). A rare case of paradoxical left sided endocarditis through patent foramen ovale. Marshall J Med.

[6] Vittala SS, Demaerschalk BM, Huettl EA, Burke RF, Chaliki HP (2011). Diagnosis of pulmonary arteriovenous malformation using a transesophageal echocardiography bubble study. Eur J Echocardiogr.

[7] Rosen P, Armstrong D (1973). Nonbacterial thrombotic endocarditis in patients with malignant neoplastic diseases. Am J Med.

[8] Lopez JA, Ross RS, Fishbein MC, Siegel RJ (1987). Nonbacterial thrombotic endocarditis: a review. Am Heart J.

